# Life Imprints: Living in a Contaminated World

**DOI:** 10.1289/ehp.1103451

**Published:** 2011-05-13

**Authors:** David Crews, Andrea C. Gore

**Affiliations:** 1 Section of Integrative Biology,; 2 Institute for Cellular and Molecular Biology, and; 3 Division of Pharmacology and Toxicology, University of Texas at Austin, Austin, Texas, USA

**Keywords:** endocrine-disrupting chemicals, epigenetics, evolutionary biology, heritable, proximate effect, ultimate effect

## Abstract

Background: The links between nature and nurture need to be redefined to accommodate anthropogenic chemical contamination. Although some local remediation of contamination has occurred, at the global level this is simply not possible. Contaminants are spread by population migration, by introduction via the food chain, and through air and water currents, even to regions that were never exposed directly to these environmental insults. In recognizing and accepting this worldwide change, we must move on and consider the types of adaptations that could occur as a consequence.

Objectives: We propose a paradigm shift in the field that integrates various disciplines involved in the study of environmental contamination to recognize that contamination is widespread and cannot be remedied at the global level.

Discussion: Greater effort must be placed on integrative and interdisciplinary studies that explicitly illuminate how the causal mechanisms and functional outcomes of related processes operate at each level of biological organization while at the same time revealing the relations among the levels.

Conclusions: To anticipate and understand the future, we must devote more study to what is likely to happen and less to what has happened. Only then will we begin to understand how ancestral environmental exposures act at both the level of the individual and the level of their descendants to influence all aspects of life history.

We are now in the third generation since the chemical revolution (circa 1940), yet the dimensions and implications of this worldwide problem have only recently begun to be recognized. We predict that changes in chemical manufacturing processes will continue to be resisted, that the development of new chemicals without adequate testing will continue unabated, and that even if regulatory changes are implemented, these efforts will be incremental at best and geographically limited. The stark fact that society must face is that on a global scale the world is contaminated and will never return to its pristine past; the successes of “spot” cleanups are newsworthy, but the unaddressed issue of environmental remediation further disturbing an ecosystem adapting to toxicity has not been addressed. Indeed, it is our contention that the best humans can do is slow the rate and nature of contamination. Although the environmental and life sciences continue to inform environmental policy and scientists, we contend that new paradigms are needed if we are to respond to this certain predicament.

The view that the study of the past is useful to predict the future is not suited for how to live in the real world of the present. An excellent example is provided by research on endocrine-disrupting chemicals (EDCs), compounds that perturb hormonal systems and that are ubiquitous, albeit heterogeneous, in the environment. Most research on EDCs today focuses on determining what, where, when, and how such compounds act at the molecular, morphological, physiological, reproductive, and neural levels. Most of this work is conducted in the laboratory under rigorous experimental control, and an ecological context is too often missing. At the other end of the spectrum, research with wildlife most often documents the consequence of contamination by EDCs at the level of the population or, occasionally, the species, but often without accurate knowledge of the nature, mixtures, and history of exposures, and in the absence of any controls. With the few exceptions for which there is clear evidence of a specific event with an identifiable chemical signature such as a toxic spill or contamination of food or water, it is often not possible to pinpoint the causes of population declines as a result of endocrine disruption (Hamlin and Guillette 2010). Human exposures are comparable to those in wildlife, because people are exposed throughout their lives, and the cause and effect and the underlying mechanisms can be difficult to ascertain. Epidemiological studies have provided some insight into potential links between exposure and the manifestation of disease, but again, there are few clear and specific causal relationships (Colborn 2004; Landrigan and Miodovnik 2011).

Only recently have studies been initiated that model real life. Starlings foraging in the winter on worms in sewage effluent filter beds receive significantly higher amounts of synthetic and natural estrogens and other EDCs than do starlings foraging on worms found in garden soil (Markman et al. 2008). During the winter, captive male starlings were fed mealworms containing ecologically relevant levels of a mixture of EDCs found in worms in contaminated sites during the winter. The next spring, both males and females were assessed for the amount and complexity of song and the size of song nuclei [higher vocal center (HVC)]. Male song and HVC volume were increased in individuals receiving the mixture; these males also showed significantly lower immune function. Females preferred the more complex song of males that had received the EDC mixture. Thus, by selecting males with more complex song, the females were also selecting males that were immunocompromised. In a multigenerational study of fathead minnows, chronic exposure (7 years) of natural populations to ecologically relevant levels of ethinyl estradiol led to near extinction (Kidd et al. 2007).

Animals (and humans) are exposed to EDCs at all stages of their life cycle, and the effects of such exposures are now known to be passed to subsequent generations. Recent research has begun to model this scenario of transgenerational effects of EDCs (Skinner et al. 2010). This is important, because we need to distinguish between those chemicals that can have permanent effects in the exposed individuals and their descendants, even in the absence of further exposure, and those compounds that have transient effects that can be mitigated or reversed through education, decontamination, and therapeutic manipulations.

Thus, a sea change in research is necessary, from simply trying to understand the specific molecular target of an EDC, to focusing instead on the consequences for the population. We are not saying that it is not vital to continue to identify EDCs, their mechanisms of disruption, or the consequences of acute exposure on the individual. However, we need to consider the future realistically in order to be able to live in our contaminated world.

The case for understanding the impact of EDCs on reproduction is enlightening because of the long-standing literature for diminished fertility and reproductive abnormalities in wildlife, laboratory animals, and human populations. Research tends to focus only on those individuals that are compromised. Nevertheless, with few exceptions, in every contaminated environment successful reproduction (viable, fertile offspring) still occurs (Crews et al. 2000). This cannot be attributed to unexposed immigrants, because many of the affected species are sedentary or nonmigratory. Thus, these reproductively successful individuals have in some way overcome the effects of local EDC contamination. Although it is possible through selection to acquire resistance to agents such as the pesticide DDT (dichlorodiphenyltrichloroethane) (e.g., Ozburn and Morrison 1962; Poonacha et al. 1973; see also Wirgin et al. 2011), which is known to act through steroid hormone receptors, it is unlikely that the successive resistant generations became insensitive to the endogenous hormones that regulate development and reproduction or they would not have been able to continue to reproduce. Evolution selects for outcomes, not mechanisms, and it is more likely that the target molecules, some of which are hormone receptors, must operate under conditions of chronic exposure to EDCs such that, over generations and during the process of genetic assimilation, novel molecular mechanisms will arise.

The distinction of ultimate versus proximate factors is long-standing in evolutionary biology (Baker 1938). In terms of heredity and environment, ultimate factors refer to how selection (natural, sexual, or artificial) gives rise to population and species differences, whereas proximate factors encompass those experiences that an individual accumulates within its life history. In relation to reproduction, ultimate factors are those environmental features that determine when young can be most efficiently raised, whereas proximate factors are those cues that enable individuals to adjust or synchronize reproductive processes so that they breed at the appropriate time. Hence, ultimate factors are responsible for the timing of breeding seasons, whereas proximate factors keep the organism synchronized with its environment. Ultimate factors operate in evolutionary time, whereas proximate factors operate in life history time. In real life, heredity (“ultimate”) and the environment (“proximate”) interact to shape the adult phenotype irrespective of the genotype.

Inherited factors predispose the individual to respond in different ways to environmental factors. This concept of environment shaping phenotype has been appreciated for centuries, before the discovery of genetics, and is exemplified by two now-popular concepts: the thrifty phenotype hypothesis (Barker 2006) and the developmental origins of health and disease (Gluckman and Hanson 2004). Genetic mutation (single or multiple genes) is clearly associated with a number of disease states. The gene × environment interaction extends to inputs from the external world, including social, biotic, and physical stimuli, as well as the maternal amniotic environment of the fetus, influencing the expression of our genes. (Interaction is defined as a mutual or reciprocal action that two or more objects have upon one another and can be additive, synergistic, or emergent in nature; Table 1.) A proof of concept is the classic example of mental retardation due to inherited phenylketonuria, which can be prevented by controlling dietary exposures (van Spronsen 2010).

**Table 1:** Types of interactions.

Interaction type	Description	Example
Additivity		When two or more factors summate (positive) or subtract (negative) in their effect.		1 + 2 = 3
Synergism		A basic principle that refers to the phenomenon that the combination of two or more factors is greater than the sum of their individual effects. For reasons other than conceptual, this is a contentious issue among toxicologists and epidemiologists, who typically deal with dosages and frequencies and often use the terms additive interaction, response addition, or dose additivity. However, we prefer the word *synergism* because of its widespread use in physics, chemistry, and biology.		1 + 2 = 5
Emergent		Originally credited to Aristotle (“the whole is something over and above its parts, and not just the sum of them all”), this concept has been elemental in philosophy and the sciences (Corning 2010). Paul Weiss (1939), perhaps the first general systems theorist in biology, believed it to be basic to all biological laws. We support the definition of Mayr (1988): “When two entities are combined at a higher level of integration, not all the properties of the new entity are necessarily a logical or predictable consequence of the properties of the components.”		Two gases combine to create a liquid

Development is a process that unfolds and reveals, with the present building on the past and setting the stage for the future. Embedded in any definition of development is the concept of emergent properties (Gilbert and Epel 2009) (Table 1). Environmental exposures can be simultaneous or temporally separated. Simultaneous exposures are most typically found when the stimuli consist of mixtures, such as chemical mixtures of similar or different properties or biological mixtures of physical, biotic, and social signals. Sequential exposures can be separated by seconds, minutes, hours, days, seasons, years, or generations, or can even skip generations, and are a fundamental property of the life cycle. Of particular relevance is the organization–activation hypothesis (Arnold 2009), originally developed in embryology and extended to endocrinology. Here the initial exposure (e.g., estrogen) early in development “primes” or organizes the target tissue to respond to a later exposure of the same hormone at a heightened level. The current “two-hit” paradigm in cancer biology, similarly, shows that early-life insult can predispose the individual to disease development in response to a second hit of an environmental exposure or even an endogenous hormone. We predict similar relationships between first hits predisposing future generations to second (or third) hits.

Another feature that must be clarified is the specificity of the interaction (Table 2). For example, an alteration in the germline caused by EDC exposure can affect the individual’s response to challenges in its life history. These challenges can be specific; for example, exposure to an estrogenic EDC may reprogram the germline such that exposed individuals in adulthood, and their offspring, have altered responses to estrogenic but not other types of compounds. In this scenario, the heritable factor defines the type(s) of challenges that the individuals are sensitive to. By contrast, there may be a more general reprogramming caused by EDCs, such that exposed individuals and their offspring have altered responses to environmental (proximate) stimuli that go beyond a single system (e.g., estrogenic EDCs) and affect a broader array of responses, including stress responsiveness, as we use as an example below.

**Table 2:** Examples of epigenetic modifications and their interactions with the environment (for illustrative purposes only).

Effect	Germline dependent	Context dependent	Germline + context
General		An antiandrogenic EDC alters DNA methylation in the male germline in a manner that alters the organism’s responses to other environmental EDCs beyond the androgen pathway, in a heritable manner.		An estrogenic EDC alters DNA methylation independently of the male germline, such that offspring will not have the modified epigenetic trait unless they, too, are exposed to the estrogenic EDC during the same critical period of development. Additionally, responses to other environmental EDCs would be altered but only in the presence of the original insult.		An antiandrogenic EDC alters DNA methylation in the male germline in a manner that alters the organism’s responses to other environmental EDCs beyond the androgen pathway. In addition, exposure to an estrogenic EDC that does not affect the germline causes epigenetic changes that affect sensitivity to other EDCs beyond the estrogen pathway. The germline-dependent modifications to the epigenome and to the sensitivity to a variety of EDCs would be inherited by the offspring; the germline-independent traits would not be inherited.
Specific		An antiandrogenic EDC alters DNA methylation in the male germline in a manner that alters the organism’s responses to other environmental antiandrogens, in a heritable manner. Responses to other classes of EDCs are unaltered.		An estrogenic EDC alters DNA methylation independently of the male germline, such that offspring will not have the modified epigenetic trait unless they too are exposed to the estrogenic EDC during the same critical period of development. Responses to other classes of EDCs are unaltered.		An antiandrogenic EDC alters DNA methylation in the male germline in a manner that alters the organism’s responses to other environmental antiandrogens. In addition, exposure to an estrogenic EDC that does not affect the germline causes other epigenetic changes that affect further estrogenic sensitivity. The germline-dependent epigenetic modifications and sensitivity to antiandrogens would be inherited by the offspring; the germline-independent epigenetic modifications and sensitivity to estrogens would not be inherited.

Finally, interactions between ultimate and proximate factors can engage distinctly different epigenetic mechanisms (Table 2). In the real world, environmental factors that bring about an epigenetic modification may simply continue to persist. For example, if the diet, behavior, or a toxic environmental exposure such as lead continues across generations, the epigenetic modification will manifest in each generation, independently of germline transmission of the modified trait. Such environmentally induced epigenetic state(s) can be reversed by removal or alteration of the factor, addition of a different environmental factor, or emigration from the contaminated site. This mitotic transgenerational effect is termed “context-dependent” epigenetic change (Crews 2008; Walker and Gore 2011) because it requires continued exposure to the environmental insult. Alternatively, the epigenetic modification may occur when the change in the epigenome is incorporated into the germline, a process termed “germline-dependent” epigenetic change. In this manner, the effect manifests in each generation even in the absence of the causative agent. Context-dependent epigenetic modification is fundamentally different from germline-dependent epigenetic modification. Although both have been attributed with “transgenerational” properties, only in the latter (germline) instance will the trait be passed to the next generation even in the absence of any continued exposures or stimuli.

Ongoing research is examining how these two forms of epigenetic modification, one carried in the germline and the other contained in the context of life history, might interact to shape morphology, physiology, brain metabolism, neurogenomics, and behavior. For example, prenatal exposure to the fungicide vinclozolin causes adverse changes in male fertility, promotes adult onset of disease, and alters brain and behavior across generations (Anway et al. 2005; Crews et al. 2007). Because these phenotypes occur in the absence of further exposure of the descendants to vinclozolin, this exemplifies germline-dependent inheritance of complex traits. How these animals respond to proximate stimuli during a critical life history stage, such as stress during adolescence, would illustrate the interplay of ultimate and proximate epigenetics. We propose a system of categorizing epigenetic phenomena as germline dependent, context dependent, or both, taking into consideration whether effects are general or specific (Table 2). Although studies of germline- and context-dependent epigenetic modifications are useful and informative in their own right, only when studies combine the two types will the real world be reflected and understood.

By its very nature, endocrine disruption influences all levels of biological organization. Accordingly, future studies should strive to be integrative and interdisciplinary, with their goal being to document effects at the genetic, epigenetic, physiological, behavioral, and neural levels and to illuminate how the causal mechanisms and functional outcomes of related processes operate at each level of biological organization. At the same time, such studies should illuminate the relations among the levels to “bring the phenotype into being” (Waddington 1942). This means that concepts from different fields of biology must be used, and it would be a mistake for any particular field to try to redefine, or let preconceptions cause one to dismiss, valid constructs because they are unfamiliar. We have discussed some of these concepts, particularly proximate versus ultimate factors in evolutionary biology; genetic, environmental, and epigenetic effects in developmental biology; and additive and synergistic interactions in physiology. Although these concepts tend to be used by practitioners in their respective fields, they rarely have crossed into different disciplines, even though they are standard in their own discipline of origin. That is, proximate and ultimate causation is a classic distinction in evolutionary biology, ethology, and organismic biology, but comparable concepts are not commonplace in molecular biology. Similarly, standard concepts in behavioral development or epidemiology are often foreign to behavioral ecology or molecular biology. Additionally, the meaning of the word “epigenetics” differs depending upon discipline, with some fields specifying molecular epigenetics and others “molar” epigenetics (Crews 2008). Finally, nonmonotonic dose–response curves, responsiveness to low doses well below “safe” no observable adverse effect levels (NOAELs) (Sheehan 2006; vom Saal et al. 2010), and synergistic interactions are well understood in endocrine physiology (Calabrese 2010; McEwen 2010), but these terms tend to be avoided or the concepts even dismissed in environmental toxicology. This highlights the need to form collaborations and to formulate a common vocabulary to enable cross talk and cross-fertilization.
